# Supplementary material for: Pacing during an ultramarathon running event in hilly terrain
Source: PeerJ. 2016 Oct 27;4:e2591. doi: 10.7717/peerj.2591 (PMC5088578; doi:10.7717/peerj.2591)
Supplement: Supplemental Information 1 [file peerj-04-2591-s001.pdf]

Article: Pacing during an ultramarathon running event in hilly terrain

Authors: Hugo A. Kerhervé, Tom Cole-Hunter, Aaron N. Wiegand, Colin Solomon

Supporting document: Prompts used for statistical analyses in R package

---

```
##################################
##set up workspace##
##################################
setwd("D:/6. Articles, projects and reviews/GPS - Great North Walk AUS/R files/")
#
#R library for descriptive stats
library(pastecs)
#R library for partial eta-squared
library(lsr)
#R library for posthoc LSD
library(agricolae)
#R library to perform Dunn's method (posthoc Kruskal-Wallis)
library(FSA)
#
#call dataset
data.h <- read.csv("GNW_relspeed.csv")
#
stat.desc(data.h)
#
###################################
##Test Normality + Homogeneity##
###################################
## speed on all gradients ##
ks.test(data.h$speed_173, data.h$speed_103, "pnorm", 1, 2)
var.test(data.h$speed_173, data.h$speed_103)
## speed on level ##
ks.test(data.h$speed_lev_173, data.h$speed_lev_103, "pnorm", 1, 2)
var.test(data.h$speed_lev_173, data.h$speed_lev_103)
## speed on UH ##
ks.test(data.h$speed_UH_173, data.h$speed_UH_103, "pnorm", 1, 2)
var.test(data.h$speed_UH_173, data.h$speed_UH_103)
## speed on DH ##
ks.test(data.h$speed_DH_173, data.h$speed_DH_103, "pnorm", 1, 2)
var.test(data.h$speed_DH_173, data.h$speed_DH_103)
#
###################################
##Test Differences in Means##
###################################
t.test(data.h$speed_173, data.h$speed_103, paired=F, data=data.h)
t.test(data.h$speed_lev_173, data.h$speed_lev_103, paired=F, data=data.h)
t.test(data.h$speed_UH_173, data.h$speed_UH_103, paired=F, data=data.h)
t.test(data.h$speed_DH_173, data.h$speed_DH_103, paired=F, data=data.h)
#
###################################
##Test Dynamics of speed as a function of distance##
###################################
#
```

```
## Kruskal-Wallis test ##
#
kruskal.test(speed_lev ~ dist3, data = data.h)
kruskal.test(speed_UH ~ dist3, data = data.h)
kruskal.test(speed_DH ~ dist3, data = data.h)
#
## posthoc Dunn's test ##
dunnTest(speed_lev ~ dist3, data = data.h)
dunnTest(speed_UH ~ dist3, data = data.h)
dunnTest(speed_DH ~ dist3, data = data.h)
#
#
##################################
##Test Correlations##
##################################
#
#call dataset
data.c <- read.csv("GNW_correlations.csv")
#
##Test Normality with Shapiro-Wilk##
shapiro.test(data.c$stoptime)
shapiro.test(data.c$loss)
shapiro.test(data.c$CV)
#
#
##Test Correlations##
#all participants
cor.test(data.c$Savg, data.c$stoptime)
cor.test(data.c$Savg, data.c$loss)
cor.test(data.c$Savg, data.c$CV)
#
#173-km participants
cor.test(data.c$Savg_173, data.c$stoptime_173)
cor.test(data.c$Savg_173, data.c$loss_173)
cor.test(data.c$Savg_173, data.c$CV_173)
#
#103-km participants
cor.test(data.c$Savg_103, data.c$stoptime_103)
cor.test(data.c$Savg_103, data.c$loss_103)
cor.test(data.c$Savg_103, data.c$CV_103)
```
