# Supplementary material for: Pacing during an ultramarathon running event in hilly terrain
Source: PeerJ. 2016 Oct 27;4:e2591. doi: 10.7717/peerj.2591 (PMC5088578; doi:10.7717/peerj.2591)
Supplement: Supplemental Information 2 [file peerj-04-2591-s002.pdf]

Article: Pacing during an ultramarathon running event in hilly terrain

Authors: Hugo A. Kerhervé, Tom Cole-Hunter, Aaron N. Wiegand, Colin Solomon

Supporting document: Summary of statistical tests

CSV datasets
GNW_relspeed Speed of all participants at all gradients (overall, level, DH, UH).
GNW_correlations Average speed and speed loss, speed variability and time stopped.

Differences between participants groups.

1. Descriptive statistics of speed in the two participants groups.

**ABSOLUTE SPEED**	All participants	173-km	103-km
n	15	10	5
Mean speed (km/h): average *(SD)*	5.66 *(1.43)*	5.68 *(1.51)*	5.91 *(1.48)*
Mean speed (km/h): level *(SD)*	5.52 *(1.46)*	5.48 *(1.53)*	5.68 *(1.64)*
Mean speed (km/h): downhill *(SD)*	6.34 *(1.51)*	6.43 *(1.64)*	6.62 *(1.40)*
Mean speed (km/h): uphill *(SD)*	5.11 *(1.05)*	5.12 *(1.03)*	5.42 *(1.22)*

**RELATIVE SPEED**	All participants	173-km	103-km
n	15	10	5
Mean speed (km/h): level *(SD)*	106.8 *(34.2)*	105.5 *(32.7)*	111.0 *(39.0)*
Mean speed (km/h): downhill *(SD)*	122.6 *(32.3)*	122.3 *(32.6)*	123.6 *(31.8)*
Mean speed (km/h): uphill *(SD)*	98.0 *(21.8)*	96.9 *(20.7)*	101.7 *(24.8)*

2. Test normality and homogeneity of variance between participant groups

H_0 = date are normal / variances are homogenous

```
> #speed all gradients
> ks.test(data.h$speed_173, data.h$speed_103, "pnorm", 1, 2)
        Two-sample Kolmogorov-Smirnov test
    data:  data.h$speed_173 and data.h$speed_103
    D = 0.085, p-value = 0.2698                                        NS
> var.test(data.h$speed_173, data.h$speed_103)
        F test to compare two variances
    data:  data.h$speed_173 and data.h$speed_103
    F = 0.86158, num df = 599, denom df = 179, p-value = 0.2027        NS
    alternative hypothesis: true ratio of variances is not equal to 1
    95 percent confidence interval: 0.6747979 1.0833535
    sample estimates: ratio of variances     0.861581
> #speed on level
> ks.test(data.h$speed_lev_173, data.h$speed_lev_103, "pnorm", 1, 2)
        Two-sample Kolmogorov-Smirnov test
    data:  data.h$speed_lev_173 and data.h$speed_lev_103
    D = 0.14, p-value = 0.3261                                         NS
> var.test(data.h$speed_lev_173, data.h$speed_lev_103)
        F test to compare two variances
    data:  data.h$speed_lev_173 and data.h$speed_lev_103
    F = 0.70244, num df = 199, denom df = 59, p-value = 0.07659        NS
    alternative hypothesis: true ratio of variances is not equal to 1
    95 percent confidence interval: 0.4535352 1.0380745
    sample estimates: ratio of variances     0.7024427
> #speed on UH
> ks.test(data.h$speed_UH_173, data.h$speed_UH_103, "pnorm", 1, 2)
        Two-sample Kolmogorov-Smirnov test
```

```
        data:  data.h$speed_UH_173 and data.h$speed_UH_103
        D = 0.17667, p-value = 0.1121                                        NS
> var.test(data.h$speed_UH_173, data.h$speed_UH_103)
            F test to compare two variances
        data:  data.h$speed_UH_173 and data.h$speed_UH_103
        F = 0.69704, num df = 199, denom df = 59, p-value = 0.07032          NS
        alternative hypothesis: true ratio of variances is not equal to 1
        95 percent confidence interval: 0.4500463 1.0300888
        sample estimates: ratio of variances        0.697039
> #speed on DH
> ks.test(data.h$speed_DH_173, data.h$speed_DH_103, "pnorm", 1, 2)
            Two-sample Kolmogorov-Smirnov test
        data:  data.h$speed_DH_173 and data.h$speed_DH_103
        D = 0.096667, p-value = 0.7815                                       NS
> var.test(data.h$speed_DH_173, data.h$speed_DH_103)
            F test to compare two variances
        data:  data.h$speed_DH_173 and data.h$speed_DH_103
        F = 1.0545, num df = 199, denom df = 59, p-value = 0.8311            NS
        alternative hypothesis: true ratio of variances is not equal to 1
        95 percent confidence interval: 0.6808275 1.5583126
        sample estimates: ratio of variances        1.054477
```

3. Test differences in means between participant groups

   H_0 = means are not different

```
> t.test(data.h$speed_173, data.h$speed_103, paired=F, data=data.h)
            Welch Two Sample t-test
        data:  data.h$speed_173 and data.h$speed_103
        t = -1.3795, df = 277.94, p-value = 0.1689                           NS
        alternative hypothesis: true difference in means is not equal to 0
        95 percent confidence interval: -9.354012  1.645781
        sample estimates: mean of x mean of y:  108.2534  112.1075
> t.test(data.h$speed_lev_173, data.h$speed_lev_103, paired=F, data=data.h)
            Welch Two Sample t-test
        data:  data.h$speed_lev_173 and data.h$speed_lev_103
        t = -0.99368, df = 85.363, p-value = 0.3232                          NS
        alternative hypothesis: true difference in means is not equal to 0
        95 percent confidence interval: -16.506104   5.504952
        sample estimates: mean of x mean of y:  105.5438  111.0444
> t.test(data.h$speed_UH_173, data.h$speed_UH_103, paired=F, data=data.h)
            Welch Two Sample t-test
        data:  data.h$speed_UH_173 and data.h$speed_UH_103
        t = -1.3671, df = 85.151, p-value = 0.1752                           NS
        alternative hypothesis: true difference in means is not equal to 0
        95 percent confidence interval: -11.803943   2.185043
        sample estimates: mean of x mean of y:  96.87732 101.68677
> t.test(data.h$speed_DH_173, data.h$speed_DH_103, paired=F, data=data.h)
            Welch Two Sample t-test
        data:  data.h$speed_DH_173 and data.h$speed_DH_103
        t = -0.26618, df = 99.287, p-value = 0.7906                          NS
        alternative hypothesis: true difference in means is not equal to 0
        95 percent confidence interval:
         -10.587159   8.082517
        sample estimates: mean of x mean of y: 122.3389  123.5912
```

Dynamics of speed within each gradient categories.

1. One-way ANOVA on ranks (Kruskal-Wallis).

   H_0 = speed does not change as a function of distance.

```
> kruskal.test(speed_lev ~ dist3, data = data.h)
        Kruskal-Wallis rank sum test
    data:  speed_lev by dist3
    Kruskal-Wallis chi-squared = 159.86, df = 19, p-value < 2.2e-16        *
> kruskal.test(speed_DH ~ dist3, data = data.h)
        Kruskal-Wallis rank sum test
    data:  speed_DH by dist3
    Kruskal-Wallis chi-squared = 188.19, df = 19, p-value < 2.2e-16        *
> kruskal.test(speed_UH ~ dist3, data = data.h)
        Kruskal-Wallis rank sum test
    data:  speed_UH by dist3
    Kruskal-Wallis chi-squared = 211.31, df = 19, p-value < 2.2e-16        *
```

2. <u>Perform Dunn's test of multiple comparisons.</u>

```
> dunnTest(speed_lev ~ dist3, data = data.h)
    Dunn (1964) Kruskal-Wallis multiple comparison
      p-values adjusted with the Holm method.

      Comparison        Z     P.unadj       P.adj
    16     5 - 35  4.14192370 3.444049e-05 5.579360e-03        *
    22     5 - 40  5.42140424 5.913267e-08 1.064388e-05        *
    29     5 - 45  4.59836078 4.258281e-06 7.239078e-04        *
    37     5 - 50  8.03135030 9.640565e-16 1.831707e-13        *
    56     5 - 60  3.52282022 4.269809e-04 6.404713e-02        t
    67     5 - 65  5.26815881 1.377989e-07 2.439040e-05        *
    79     5 - 70  5.42450977 5.811381e-08 1.051860e-05        *
    92     5 - 75  3.88054402 1.042231e-04 1.636302e-02        *
    106    5 - 80  6.03036975 1.635850e-09 3.042681e-07        *
    137    5 - 90  6.29421199 3.089653e-10 5.777652e-08        *
    154    5 - 95  5.58086073 2.393312e-08 4.403695e-06        *
    172    5 - 100  3.47012274 5.202206e-04 7.699265e-02       t

    23     10 - 40  4.51095794 6.453552e-06 1.084197e-03       *
    30     10 - 45  3.68791448 2.260996e-04 3.459324e-02       *
    38     10 - 50  7.12090400 1.072215e-12 2.026486e-10       *
    68     10 - 65  4.45383088 8.435155e-06 1.400236e-03       *
    80     10 - 70  4.61018184 4.023169e-06 6.879619e-04       *
    107    10 - 80  5.21604182 1.827869e-07 3.217050e-05       *
    138    10 - 90  5.47988407 4.256046e-08 7.788565e-06       *
    155    10 - 95  4.76653280 1.874232e-06 3.261164e-04       *

    39     15 - 50  4.62992292 3.658019e-06 6.291792e-04       *

    40     20 - 50  5.46996138 4.501337e-08 8.192433e-06       *
    109    20 - 80  3.73939384 1.844645e-04 2.859200e-02       *
    140    20 - 90  4.00323609 6.248189e-05 9.997103e-03       *

    26     25 - 40  4.40898795 1.038548e-05 1.713604e-03       *
    33     25 - 45  3.58594450 3.358604e-04 5.071492e-02       *
    41     25 - 50  7.01893402 2.235673e-12 4.203066e-10       *
    71     25 - 65  4.36262615 1.285104e-05 2.107571e-03       *
    83     25 - 70  4.51897711 6.213911e-06 1.050151e-03       *
    110    25 - 80  5.12483709 2.977951e-07 5.211415e-05       *
    141    25 - 90  5.38867934 7.097731e-08 1.270494e-05       *
    158    25 - 95  4.67532807 2.934841e-06 5.077274e-04       *

    42     30 - 50  5.67147349 1.415745e-08 2.619128e-06       *
    111    30 - 80  3.91963176 8.868437e-05 1.410081e-02       *
    142    30 - 90  4.18347401 2.870879e-05 4.679533e-03       *
```

```
43    35 - 50  3.88942660 1.004814e-04 1.587605e-02    *

45    45 - 50  3.43298952 5.969652e-04 8.775388e-02    *

55    50 - 55 -5.30001140 1.157955e-07 2.061159e-05    *
65    50 - 60 -4.50853008 6.527831e-06 1.090148e-03    *
130   50 - 85 -4.05861039 4.936560e-05 7.947861e-03    *
181   50 - 100 -3.71333535 2.045456e-04 3.150002e-02   *

116   55 - 80  3.58738596 3.340097e-04 5.076948e-02    *
147   55 - 90  3.85122821 1.175269e-04 1.833420e-02    *

> dunnTest(speed_UH ~ dist3, data = data.h)
Dunn (1964) Kruskal-Wallis multiple comparison
p-values adjusted with the Holm method.
   Comparison        Z      P.unadj        P.adj
2     5 - 15  5.63505564 1.750018e-08 2.975031e-06     *
16    5 - 35  5.85113489 4.882300e-09 8.592849e-07     *
22    5 - 40  6.35127340 2.135398e-10 3.950486e-08     *
29    5 - 45  5.86812989 4.407379e-09 7.801061e-07     *
37    5 - 50  6.05264701 1.424849e-09 2.550479e-07     *
67    5 - 65  3.66881876 2.436737e-04 3.289596e-02     *
79    5 - 70  6.51570919 7.234702e-11 1.352889e-08     *
92    5 - 75  6.23883770 4.408341e-10 8.067264e-08     *
106   5 - 80  4.15741551 3.218683e-05 4.731463e-03     *
137   5 - 90  6.21929382 4.993974e-10 9.089032e-08     *
154   5 - 95  7.37889679 1.596065e-13 3.032523e-11     *
172   5 - 100  3.46686543 5.265654e-04 6.740038e-02    t

80    10 - 70  3.85557129 1.154597e-04 1.581798e-02    *
93    10 - 75  3.57869980 3.453078e-04 4.523533e-02    *
138   10 - 90  3.55915593 3.720486e-04 4.799427e-02    *
155   10 - 95  4.71875890 2.372878e-06 3.749148e-04    *

6     15 - 20 -5.55493636 2.777129e-08 4.693348e-06    *
9     15 - 25 -3.77288947 1.613678e-04 2.194602e-02    *
13    15 - 30 -4.74403219 2.095055e-06 3.331138e-04    *
58    15 - 60 -3.90156588 9.557244e-05 1.318900e-02    *

19    20 - 35  5.77101562 7.879517e-09 1.363156e-06    *
25    20 - 40  6.27115412 3.583817e-10 6.594223e-08    *
32    20 - 45  5.78801062 7.122491e-09 1.239313e-06    *
40    20 - 50  5.97252773 2.336054e-09 4.158176e-07    *
70    20 - 65  3.59715790 3.217132e-04 4.246614e-02    *
82    20 - 70  6.44404833 1.163280e-10 2.163701e-08    *
95    20 - 75  6.16717684 6.952000e-10 1.258312e-07    *
109   20 - 80  4.08575465 4.393378e-05 6.326465e-03    *
140   20 - 90  6.14763297 7.864776e-10 1.415660e-07    *
157   20 - 95  7.30723594 2.726935e-13 5.153907e-11    *

20    25 - 35  3.98896873 6.636117e-05 9.290564e-03    *
26    25 - 40  4.48910723 7.152229e-06 1.087139e-03    *
33    25 - 45  4.00596372 6.176513e-05 8.708883e-03    *
41    25 - 50  4.19048084 2.783639e-05 4.119786e-03    *
83    25 - 70  4.85013714 1.233761e-06 1.974018e-04    *
96    25 - 75  4.57326564 4.801807e-06 7.394783e-04    *
141   25 - 90  4.55372177 5.270505e-06 8.063873e-04    *
158   25 - 95  5.71332474 1.107900e-08 1.894508e-06    *
```

```
21    30 - 35  4.96011145 7.045275e-07 1.134289e-04  *
27    30 - 40  5.46024995 4.754647e-08 7.987807e-06  *
34    30 - 45  4.97710644 6.454183e-07 1.052032e-04  *
42    30 - 50  5.16162356 2.448172e-07 4.015002e-05  *
84    30 - 70  5.71875359 1.073083e-08 1.845702e-06  *
97    30 - 75  5.44188210 5.272057e-08 8.804335e-06  *
142   30 - 90  5.42233823 5.882444e-08 9.764858e-06  *
159   30 - 95  6.58194120 4.643453e-11 8.729691e-09  *

62    35 - 60 -4.11764514 3.827635e-05 5.550070e-03  *

53    40 - 55 -3.91370517 9.089060e-05 1.263379e-02  *
63    40 - 60 -4.61778364 3.878603e-06 6.011835e-04  *

64    45 - 60 -4.13464013 3.555113e-05 5.190465e-03  *

55    50 - 55 -3.61507878 3.002563e-04 4.023434e-02  *
65    50 - 60 -4.31915725 1.566262e-05 2.333730e-03  *

89    55 - 70  4.33548189 1.454412e-05 2.181617e-03  *
102   55 - 75  4.05861039 4.936560e-05 7.059280e-03  *
147   55 - 90  4.03906652 5.366435e-05 7.620337e-03  *
164   55 - 95  5.19866949 2.007201e-07 3.311881e-05  *

90    60 - 70  4.96522882 6.862012e-07 1.111646e-04  *
103   60 - 75  4.68835732 2.754068e-06 4.323887e-04  *
148   60 - 90  4.66881345 3.029443e-06 4.725931e-04  *
165   60 - 95  5.82841642 5.595581e-09 9.792267e-07  *

134   70 - 85 -3.60388826 3.134919e-04 4.169442e-02  *

170   85 - 95  4.39186713 1.123814e-05 1.696959e-03  *

190   95 - 100 -3.57117971 3.553769e-04 4.619900e-02  *

> dunnTest(speed_DH ~ dist3, data = data.h)
Dunn (1964) Kruskal-Wallis multiple comparison
  p-values adjusted with the Holm method.
    Comparison          Z      P.unadj        P.adj
2     5 - 15  4.01810301 5.866855e-05 8.741614e-03  *
22    5 - 40  3.98168516 6.842838e-05 1.012740e-02  *
29    5 - 45  4.49396294 6.990980e-06 1.118557e-03  *
37    5 - 50  6.10848771 1.005796e-09 1.820491e-07  *
46    5 - 55  5.20532498 1.936574e-07 3.311541e-05  *
67    5 - 65  4.04558114 5.219348e-05 7.829022e-03  *
79    5 - 70  6.41364676 1.420792e-10 2.656881e-08  *
92    5 - 75  4.99997347 5.733820e-07 9.575480e-05  *
106   5 - 80  4.24753447 2.161359e-05 3.350107e-03  *
121   5 - 85  4.78499090 1.709951e-06 2.804319e-04  *
137   5 - 90  6.52113804 6.977593e-11 1.311787e-08  *
154   5 - 95  6.82732534 8.651235e-12 1.643735e-09  *
172   5 - 100  6.26706773 3.679102e-10 6.769547e-08  *

30    10 - 45  3.75346662 1.744058e-04 2.424240e-02  *
38    10 - 50  5.36799139 7.961835e-08 1.393321e-05  *
47    10 - 55  4.46482866 8.013285e-06 1.274112e-03  *
80    10 - 70  5.75132671 8.854581e-09 1.576115e-06  *
93    10 - 75  4.33765343 1.440120e-05 2.246587e-03  *
```

```
107   10 - 80  3.58521442 3.368014e-04 4.513138e-02    *
122   10 - 85  4.12267085 3.745046e-05 5.692471e-03    *
138   10 - 90  5.85881800 4.661733e-09 8.344501e-07    *
155   10 - 95  6.16500530 7.048078e-10 1.289798e-07    *
173   10 - 100  5.60474768 2.085586e-08 3.670631e-06   *

6     15 - 20 -3.87243160 1.077549e-04 1.562446e-02    *

25    20 - 40  3.83601375 1.250474e-04 1.775673e-02    *
32    20 - 45  4.34829153 1.372021e-05 2.154074e-03    *
40    20 - 50  5.96281631 2.479271e-09 4.462688e-07    *
49    20 - 55  5.05965358 4.200189e-07 7.098319e-05    *
70    20 - 65  3.91528868 9.029610e-05 1.318323e-02    *
82    20 - 70  6.28335429 3.313448e-10 6.129878e-08    *
95    20 - 75  4.86968101 1.117786e-06 1.844346e-04    *
109   20 - 80  4.11724200 3.834334e-05 5.789844e-03    *
124   20 - 85  4.65469843 3.244552e-06 5.223729e-04    *
140   20 - 90  6.39084557 1.649709e-10 3.068459e-08    *
157   20 - 95  6.69703288 2.126938e-11 4.019913e-09    *
175   20 - 100  6.13677526 8.421339e-10 1.532684e-07   *

41    25 - 50  4.43812223 9.074708e-06 1.433804e-03    *
50    25 - 55  3.53495950 4.078377e-04 5.424241e-02    *
83    25 - 70  4.91962645 8.670953e-07 1.439378e-04    *
96    25 - 75  3.50595317 4.549753e-04 6.005673e-02    t
141   25 - 90  5.02711774 4.979067e-07 8.364832e-05    *
158   25 - 95  5.33330504 9.644110e-08 1.678075e-05    *
176   25 - 100  4.77304743 1.814590e-06 2.957782e-04   *

42    30 - 50  4.77073862 1.835516e-06 2.973536e-04    *
51    30 - 55  3.86757589 1.099226e-04 1.582886e-02    *
84    30 - 70  5.21712759 1.817191e-07 3.125568e-05    *
97    30 - 75  3.80345431 1.426923e-04 1.997693e-02    *
126   30 - 85  3.58847173 3.326220e-04 4.490398e-02    *
142   30 - 90  5.32461888 1.011649e-07 1.750153e-05    *
159   30 - 95  5.63080618 1.793692e-08 3.174835e-06    *
177   30 - 100  5.07054856 3.966707e-07 6.743402e-05   *

85    35 - 70  3.81865509 1.341812e-04 1.891955e-02    *
143   35 - 90  3.92614638 8.631764e-05 1.268869e-02    *
160   35 - 95  4.23233368 2.312789e-05 3.561696e-03    *
178   35 - 100  3.67207607 2.405880e-04 3.296056e-02   *

90    60 - 70  3.73830807 1.852628e-04 2.556627e-02    *
165   60 - 95  4.15198666 3.296015e-05 5.042903e-03    *
148   60 - 90  3.84579936 1.201601e-04 1.718289e-02    *
183   60 - 100  3.59172905 3.284913e-04 4.467482e-02   *
```

Characteristics of pacing.

1. <u>Test for normality of distribution (Shapiro-Wilk).</u>

   H_0 = speed are normal.

```
> shapiro.test(data.c$stoptime)
        Shapiro-Wilk normality test
data:  data.c$stoptime
W = 0.88881, p-value = 0.06434        NS
> shapiro.test(data.c$loss)
        Shapiro-Wilk normality test
```

```
        data:  data.c$loss
        W = 0.9126, p-value = 0.1485                                    NS
> shapiro.test(data.c$CV)
            Shapiro-Wilk normality test
        data:  data.c$CV
        W = 0.9336, p-value = 0.3086                                    NS
```

2. <u>Test for simple correlations (Pearson's r) on all participants</u>

H_0 = data are not significantly correlated.

```
> cor.test(data.c$Savg, data.c$stoptime)
            Pearson's product-moment correlation
        data:  data.c$Savg and data.c$stoptime
        t = -1.3357, df = 13, p-value = 0.2046                          NS
        alternative hypothesis: true correlation is not equal to 0
        95 percent confidence interval:
         -0.7297834  0.2005657
        sample estimates:      cor -0.3473887
> cor.test(data.c$Savg, data.c$loss)
            Pearson's product-moment correlation
        data:  data.c$Savg and data.c$loss
        t = -0.89421, df = 13, p-value = 0.3875                         NS
        alternative hypothesis: true correlation is not equal to 0
        95 percent confidence interval:
         -0.6703226  0.3097393
        sample estimates:      cor -0.2407177
> cor.test(data.c$Savg, data.c$CV)
            Pearson's product-moment correlation
        data:  data.c$Savg and data.c$CV
        t = -0.83591, df = 13, p-value = 0.4183                         NS
        alternative hypothesis: true correlation is not equal to 0
        95 percent confidence interval:
         -0.6615722  0.3238849
        sample estimates:      cor -0.2258496
```

3. <u>Test for simple correlations on 173-km participants</u>

```
> cor.test(data.c$Savg_173, data.c$stoptime_173)
            Pearson's product-moment correlation
        data:  data.c$Savg_173 and data.c$stoptime_173
        t = -1.413, df = 8, p-value = 0.1953
        alternative hypothesis: true correlation is not equal to 0
        95 percent confidence interval:
         -0.8401366  0.2542543
        sample estimates:      cor -0.4469168
> cor.test(data.c$Savg_173, data.c$loss_173)
            Pearson's product-moment correlation
        data:  data.c$Savg_173 and data.c$loss_173
        t = 0.0019574, df = 8, p-value = 0.9985
        alternative hypothesis: true correlation is not equal to 0
        95 percent confidence interval:
         -0.6292084  0.6300438
        sample estimates:        cor  0.0006920394
> cor.test(data.c$Savg_173, data.c$CV_173)
            Pearson's product-moment correlation
        data:  data.c$Savg_173 and data.c$CV_173
        t = -0.74091, df = 8, p-value = 0.4799
        alternative hypothesis: true correlation is not equal to 0
        95 percent confidence interval:
         -0.7615281  0.4476446
        sample estimates:      cor  -0.2534031
```

4. <u>Test for simple correlations on 103-km participants</u>

```
> cor.test(data.c$Savg_103, data.c$stoptime_103)
        Pearson's product-moment correlation
    data:  data.c$Savg_103 and data.c$stoptime_103
    t = -0.25727, df = 3, p-value = 0.8136
    alternative hypothesis: true correlation is not equal to 0
    95 percent confidence interval:
     -0.9110891  0.8448587
    sample estimates:       cor -0.1469218
> cor.test(data.c$Savg_103, data.c$loss_103)
        Pearson's product-moment correlation
    data:  data.c$Savg_103 and data.c$loss_103
    t = -0.82028, df = 3, p-value = 0.4722
    alternative hypothesis: true correlation is not equal to 0
    95 percent confidence interval:
     -0.9511175  0.7298650
    sample estimates:       cor -0.4280162
> cor.test(data.c$Savg_103, data.c$CV_103)
        Pearson's product-moment correlation
    data:  data.c$Savg_103 and data.c$CV_103
    t = -0.43202, df = 3, p-value = 0.6949
    alternative hypothesis: true correlation is not equal to 0
    95 percent confidence interval:
     -0.9264610  0.8140756
    sample estimates:       cor -0.2420103
```
